# Transcriptomic and RNA Modification Landscape of Severe Fever with Thrombocytopenia Syndrome Virus Revealed by Nanopore Direct RNA Sequencing

**DOI:** 10.3390/microorganisms14040756

**Published:** 2026-03-27

**Authors:** Haowen Yuan, Bohan Zhang, Ling Qiu, Jingwan Han, Lei Jia, Xiaolin Wang, Yongjian Liu, Hanping Li, Hongling Wen, Lin Li

**Affiliations:** 1 Department of Microbiological Laboratory Technology, School of Public Health Cheeloo College of Medicine, Key Laboratory for the Prevention and Control of Emerging Infectious Diseases and Biosafety, Shandong University, Jinan 250012, China; 2 State Key Laboratory of Pathogen and Biosecurity, Academy of Military Medical Sciences, Beijing 100071, China; 3 Shandong Public Health Clinical Center, Jinan 250100, China; 4 Yellow River National Strategy Institute, Shandong University, Jinan 250012, China; 5 Shandong Provincial Key Laboratory of Intelligent Monitoring, Early Warning, Prevention and Control for Infectious Diseases, Jinan 250100, China

**Keywords:** severe fever with thrombocytopenia syndrome, SFTS virus (SFTSV), Oxford Nanopore direct RNA sequencing, viral transcriptome, epitranscriptome

## Abstract

Severe Fever with Thrombocytopenia Syndrome (SFTS) is caused by the SFTS virus (SFTSV) and is associated with a high mortality rate. Although previous studies have reported RNA modifications such as m6A on SFTSV RNA, an integrated analysis of native viral transcript architecture and multiple RNA modification types within infected cells remains lacking. Here, we used Oxford Nanopore direct RNA sequencing (DRS) to analyze native SFTSV RNA in infected cells, combining strand-specific alignment, isoform reconstruction through read endpoint clustering, isoform-level quantification, and signal-level modification identification using unmodified in vitro transcripts as a baseline. This approach allowed us to construct detailed maps of the L, M, and bidirectionally encoded S segments at single-molecule, isoform-level resolution. The results reveal a “length-layering” pattern in SFTSV transcription, anchored by recurrent 3′ termination hotspots: only a few full-length transcripts dominate expression, whereas multiple reproducible truncated isoforms were associated with discrete termination windows, a pattern less consistent with random degradation alone and suggestive of regulated transcript termination. At the single-nucleotide level, the modification landscape is predominantly Ψ (pseudouridine), followed by m^5^C (5-methylcytosine), with sparse m^6^A (N6-methyladenosine). Modification hotspots are co-located across isoforms at the same genomic coordinates, exhibiting segmental/strand asymmetry, with sharper peaks on (−) RNA. These patterns provide a testable framework and raise the possibility that transcript-boundary organization and site-constrained Ψ/m5C signals may be associated with variation in viral RNA output. More broadly, isoform proportions around termination hotspots and Ψ/m5C-enriched regions at conserved sites may serve as quantitative features for characterizing viral RNA organization and prioritizing targets for future functional investigation. Our single-molecule integrated map establishes a reproducible methodological framework for studying SFTSV RNA regulation and provides a resource for future work aimed at assessing how transcript boundaries and RNA modification patterns may relate to polymerase activity and virus–host interaction.

## 1. Introduction

In recent years, Severe Fever with Thrombocytopenia Syndrome (SFTS) has been continually spreading in East Asia, becoming a serious public health threat. This disease is caused by the SFTS virus (SFTSV), which is transmitted by ticks and belongs to the genus Bandavirus within the family Phenuiviridae of the order Bunyavirales [1,2]. Since it was first identified in China in 2009, SFTSV has caused outbreaks in several countries, including Japan, South Korea, and Vietnam [3,4,5,6]. As of now, the mortality rate of SFTS varies among different populations and regions, with the mortality rate for severe cases reaching as high as 30% [1,7,8]. Therefore, the World Health Organization (WHO) has classified it as one of the priority zoonotic diseases of major concern [9].

SFTSV is a segmented negative-strand RNA virus with S, M, and L genome segments encoding non-structural protein (NSs), nucleoprotein (NP), the glycoprotein precursor (GP), and the RNA-dependent RNA polymerase (RdRp), respectively, which are involved in key processes of the viral life cycle, including replication, assembly, budding, and evasion of host immunity [10,11,12,13]. Clinical studies have shown that SFTSV can induce a range of systemic inflammatory responses, cytokine storms, and immune system dysregulation [14,15,16,17]. However, the transcriptional and epitranscriptomic features of SFTSV RNA in infected cells remain incompletely understood. A systematic characterization of SFTSV RNA architecture and modification patterns in infected cells would therefore improve our understanding of viral RNA biology and provide a framework for linking viral RNA features to infection-associated phenotypes.

During the process of viral infection, viral RNA serves not only as the core template for replication and expression but also carries various epitranscriptomic modifications that can regulate the viral life cycle and its interaction with the host immune system [18,19]. In recent years, RNA epitranscriptomic modifications have been recognized as important factors in regulating viral replication and host immune responses. Modifications such as N6-methyladenosine (m^6^A) have been extensively studied and shown to influence the life cycles of various viruses, including HIV, HBV, ZIKV, and SARS-CoV-2, by modulating RNA stability, splicing, nuclear export, and translation efficiency [20,21,22,23]. In addition to m6A, other modifications such as 5-methylcytidine (m5C), pseudouridine (Ψ), and inosine (I) may also play potential roles in viral infection, potentially involving the regulation of viral RNA structure, evasion of immune recognition, and interactions with host proteins [24,25,26,27]. Notably, epitranscriptomic regulation has also been increasingly recognized in negative-sense RNA viruses. Studies in influenza A virus and other negative-sense RNA viruses have suggested that RNA modifications can affect viral gene expression, replication, and host interaction, while direct RNA sequencing analyses of RSV have demonstrated marked transcript heterogeneity and complex transcriptional outputs. Previous studies have reported m6A modification on SFTSV RNA and implicated it in viral infection. Nevertheless, a systematic analysis based on native viral RNA molecules is still lacking, especially one that simultaneously resolves transcript architecture, multiple modification types, and their strand-specific distribution during infection.

Current mainstream RNA sequencing methods perform well in detecting changes in transcript abundance, but due to their reliance on cDNA synthesis steps, the modification information present in natural RNA is often disrupted during library preparation, making it challenging to accurately analyze RNA modification characteristics [28,29]. Direct RNA sequencing (DRS) using Nanopore technology, as a third-generation sequencing approach, allows for the direct reading of natural RNA molecules without reverse transcription, enabling the in situ capture of RNA modification signals [30,31]. This technique is particularly suitable for studying the highly dynamic and complex transcriptomes and epitranscriptomes in the context of viral infections. Furthermore, DRS can simultaneously resolve transcript structures, base modifications, and poly(A) tail lengths within the same read, providing a novel technical avenue for in-depth investigation of the interaction mechanisms between viruses and their hosts [32,33].

In this study, we isolated an SFTSV strain from clinical samples and established a cell-based infection model. Vero cells were selected because they are highly permissive to SFTSV infection and support the recovery of sufficient intracellular viral RNA for DRS analysis. We nevertheless acknowledge that RNA modification patterns may vary across host cell types, and our findings should therefore be interpreted within the context of this Vero-cell-based infection model. We then applied DRS to native viral RNA molecules present in infected cells, enabling simultaneous characterization of transcript structural features and candidate RNA modification signals. Through integrated analysis of transcript architecture and modification-associated patterns, we generated an intracellular map of SFTSV RNA features under the sampled infection condition, with particular emphasis on transcript abundance, structural isoforms, and the genomic distribution of candidate RNA modifications. This work expands the current view of SFTSV RNA biology and provides a technical framework for investigating how transcript organization and RNA modifications may contribute to viral replication and host interaction.

## 2. Materials and Methods

### 2.1. Virus Isolation from Serum of a SFTS Patient

A serum sample from a severe SFTS patient was collected during the acute phase of hospitalization at the Department of Infectious Diseases, Shandong Public Health Clinical Center. Upon admission, the patient exhibited typical clinical features such as high fever, thrombocytopenia, and leukopenia. Diagnosis of SFTSV infection was confirmed through routine hematological examinations combined with real-time fluorescence quantitative reverse transcription PCR (qRT-PCR) detection using SFTSV-specific probes. This process utilized a commercial kit (Daan Gene, Guangzhou, China), strictly following the manufacturer’s instructions. The patient’s basic information, clinical manifestations, and primary laboratory test results were meticulously recorded for subsequent analysis.

The SFTSV RNA-positive serum sample was used for blind passage virus isolation. Briefly, Vero cells were seeded in a six-well plate and incubated for one day. When they reached 40% confluence, they were incubated at 37 °C with 50 μL of serum diluted in Dulbecco’s Modified Eagle Medium (DMEM; Sigma, USA) containing 2% fetal bovine serum (FBS; Gibco, Australia) for one hour. The supernatant was then replaced with fresh DMEM containing 2% FBS, penicillin (50–100 IU/mL), and streptomycin (50–100 μg/mL), followed by a 5-day incubation at 37 °C. The supernatants from each sample were harvested and used to incubate with new healthy cells. After three passages, the supernatants were collected from each sample, centrifuged (5000× g, 5 min) to remove cell debris, and stored as virus stock at −80 °C for further analysis.

To further verify the isolated virus, culture supernatants from infected cells were subjected to transmission electron microscopy (TEM). Briefly, virus-containing material was prepared from infected cultures, applied to grids, negatively stained, and observed under a transmission electron microscope to identify typical virion morphology.

For phylogenetic analysis, the nucleotide sequences of the L, M, and S segments generated in this study were compared with representative SFTSV sequences retrieved from GenBank. After sequence alignment, maximum-likelihood phylogenetic trees were constructed using IQ-TREE, and branch support was assessed by bootstrap analysis.

### 2.2. Extraction and Preparation of SFTSV RNA

First, the infected Vero cells were collected for the extraction of total RNA from SFTSV. After reaching an appropriate state of infection (72 h post-infection), the cells were recovered by low-temperature centrifugation, followed by total RNA extraction according to the instructions of the RNA extraction kit (TaKaRa, Takara Bio Inc., Kusatsu, Shiga, Japan,, cat #9767). To enrich viral RNA for subsequent direct RNA sequencing (DRS), host rRNA was removed from total RNA using the Ribo-off^®^ rRNA Depletion Kit (Vazyme, Nanjing, China N406) following the manufacturer’s protocol. The depletion targeted both cytoplasmic rRNAs (28S, 18S, 5.8S, and 5S) and mitochondrial rRNAs (16S and 12S). rRNA depletion was performed before enzymatic poly(A) tailing, and the resulting rRNA-reduced RNA fraction was then used for downstream DRS library preparation. Although we did not perform a dedicated quantitative assessment of depletion efficiency, this step was included to reduce host rRNA background and improve viral RNA representation in the final dataset.

Next, a poly(A) tail was added to the 3′ end of the RNA. The poly(A) tailing reaction was conducted in a 20 μL reaction system containing 1–10 μg RNA, 2 μL reaction buffer, 2 μL of 10 mM ATP, 1 μL of E. coli-derived Poly(A) Polymerase (New England Biolabs), and 0.5 μL of RNaseOUT inhibitor (Invitrogen, USA). The reaction was carried out at 37 °C for 1.5 min, followed by the addition of an equal volume of 0.8× magnetic beads, and incubated at room temperature for 5 min. Then, the mixture was washed twice with 80% ethanol, and after air-drying, the RNA was eluted with nuclease-free water. We did not perform a dedicated evaluation of artificial poly(A) tail length or tailing efficiency, nor did we separately assess its effect on read length distribution.

### 2.3. In Vitro Transcription Workflow

In this study, total RNA was first extracted from Vero cells infected with SFTSV (using 0.5 µg), and specific primers designed for the viral sequences were used for the reverse transcription reaction, employing SuperScript IV reverse transcriptase (Invitrogen, USA). Subsequently, PCR amplification of each segment was performed using Q5 High-Fidelity DNA Polymerase (NEB, Ipswich, MA, USA, M0491S) to obtain the template DNA required for in vitro transcription. The primers used were the corresponding virus-specific PCR primer pairs.

The PCR products were purified using an agarose gel electrophoresis purification kit (Wizard^®^ SV Gel and PCR Clean-Up System, Promega, Madison, WI, USA, A9282), followed by in vitro transcription using the T7 High Yield RNA Transcription Kit (Vazyme, Nanjing, China, TR101). Finally, the resulting RNA products were purified using the Monarch^®^ Spin RNA Cleanup Kit (NEB, Ipswich, MA, USA, T2050L). The oligonucleotide sequences utilized are detailed in Supplementary Table S1 and Figure S1.

### 2.4. Nanopore DRS and Data Processing

To obtain the raw sequence information of viral transcripts in SFTSV-infected samples, the Direct RNA Sequencing (DRS) platform from Oxford Nanopore Technologies was used for library construction and sequencing. RNA libraries were prepared following the standard protocol provided by ONT using the direct RNA sequencing kit (Oxford Nanopore, Oxford, UK, SQK-RNA004), which includes an optional reverse transcription step to enhance adapter ligation efficiency and template stability. Sequencing was conducted on the MinION MK1C platform (Oxford Nanopore, Oxford, UK) using a flow cell (Oxford Nanopore, UK, FLO-MIN004RA) for data acquisition.

The raw electrical signal data were output in multichannel Pod5 format and base-called using the Dorado basecaller (v0.9.5) with the configuration file set to rna004_130bps_sup@v5.1.0, ensuring operation in a super-accurate mode optimized for DRS. The base-called results were saved in the standard FASTQ format. For further analysis of sequencing performance, the Pomoxis toolkit (v0.3.6) was utilized to extract basic characteristics of individual reads, summarizing information such as sequence length distribution, average quality scores, and sequencing error rates.

Sequencing reads were aligned to the viral reference genome using minimap2 (v2.28) [34] with nanopore-specific parameter settings “-ax map-ont”. The alignment results were output in SAM format and subsequently converted, sorted, and indexed using SAMtools (v1.7) to generate standardized BAM files required for subsequent analyses [35]. The entire workflow ensures quality control, retention of structural information, and compatibility with subsequent transcript reconstruction and modification analysis, providing a high-quality raw data foundation for studying the SFTSV transcriptome at a comprehensive level.

### 2.5. Transcriptome Analysis

The Nanopore Direct RNA Sequencing (DRS) data were aligned to the SFTSV reference genome using minimap2 (v2.28) (-t 16 -ax map-ont --MD -k14), focusing the analysis on viral segments. Given the bunyavirus genome lacks splicing, only primary alignments were retained, removing secondary/supplementary alignments and records containing “N” in their CIGAR strings. For each alignment, the start at position 0, the end in a half-open interval, and the strand direction were extracted and grouped by (segment, strand).

Using Nanopore direct RNA sequencing alignments (three biological replicates, BAM files), we inferred SFTSV transcript candidates directly from read-to-reference alignment endpoints. Primary alignments were parsed with pysam and converted to 1-based inclusive coordinates: for reads mapped to the positive strand, the reference-aligned start and end were defined as the 5′ and 3′ endpoints, respectively, whereas the definitions were reversed for negative-strand reads. To reduce spurious termini, reads were filtered by alignment quality and length (MAPQ ≥ 20; aligned reference length ≥ 200 nt). To reflect bunyavirus transcription features, we applied hierarchical endpoint clustering with termination as the primary axis. Within each segment and strand, 3′ endpoints were summarized by peak calling, and the top three termination peaks were retained; reads falling within ±10 nt of a peak were assigned to a termination-site (TES) family. Within each TES family, 5′ endpoints were further summarized to capture start-site heterogeneity: up to eight start-site peaks were identified and expanded to ±60 nt windows to define start-site (TSS) subtypes. Combined (TSS, TES) pairs were treated as transcript candidates. For each candidate, per-replicate read support was quantified and replicate-aware filtering was applied, requiring support in at least 2/3 replicates above a threshold, a minimum total support, and excluding candidates dominated by a single replicate (maximum fraction ≤ 0.8). For reporting, the top three candidates by total support were retained within each TES family. The pipeline outputs candidate tables, GTF annotations (one transcript and one exon per candidate), and FASTA sequences extracted from the corresponding reference intervals.

To construct a high-confidence extended SFTSV transcriptome annotation set, suspected artifacts were eliminated on cross-sample consensus, retaining only new subtypes that were single-exon, strand-specific, and with endpoints residing in high-density endpoint clusters, before merging with reference annotations to generate the extended annotation. Subsequently, reads from each sample were realigned to this extended transcriptome and quantified at the transcript level using NanoCount (v1.1.0) (--extra_tx_info --min_alignment_length 30 --min_query_fraction_aligned 0.3 --max_dist_3_prime 100 --sec_scoring_threshold 0.8) [36]. Unless otherwise specified, all other parameters were kept at default settings.

### 2.6. Epitranscriptome Analysis

After performing direct RNA sequencing of full-length SFTSV RNA samples using the Oxford Nanopore platform, the Modkit (v0.3.2) was used for base-level modification detection and analysis of the current signal. Initially, the basecalled BAM file was input into the modkit align module alongside the SFTSV reference genome (GenBank accession: HQ141595.1, HQ141596.1, HQ141597.1) to generate a modBAM file with annotated modifications. Concurrently, in vitro transcribed (IVT) RNA was constructed as a control sample to serve as an unmodified reference for background signal correction.

Subsequently, the modkit pileup and modkit extract calls modules were employed to detect m6A, m5C, and Ψ modifications by setting the modification codes --mod-code as a (m^6^A), c (m^5^C), 17802 (Ψ), and specifying the corresponding reference bases. The default model was used for current signal modeling during the detection process, producing modification probability values (mod_prob) and recording modification frequencies at each site in BED format.

To enhance the specificity and confidence of modification identification, we further used modkit dmr to compare the modBAM results of Sample RNA with IVT RNA, retaining only those sites where the modification probability was significantly higher in the native sample than in the IVT as credible candidate modifications. Credible modification sites must meet the following criteria: (1) Coverage ≥ 20×; (2) Sample RNA modification ratio > 20%; (3) Difference in modification frequency between Sample RNA and IVT, d ≥ 5%. These thresholds were empirically selected to provide a conservative balance between sensitivity and specificity. Specifically, a minimum coverage of 20× was required to reduce instability caused by low-read stochastic variation; a sample modification ratio >20% was used to exclude very low-frequency signals that are more susceptible to background noise; and a minimum difference of 5% between native sample RNA and IVT RNA was applied to enrich for sites showing reproducible signal elevation over the unmodified control background. This comparative strategy was intended to improve confidence in candidate modification calls derived from nanopore current signals. Unless otherwise specified, all other parameters were run with default settings.

In addition, to explore the spatial distribution patterns of modifications on the viral genome, we conducted motif enrichment analysis of the ±10 bp regions upstream and downstream of credible modification sites using the modkit motif search tool and identified modification-related sequence features using the MEME tool.

## 3. Results

### 3.1. Isolation and Identification of SFTSV Based on Patient Serum

**Figure 1:**
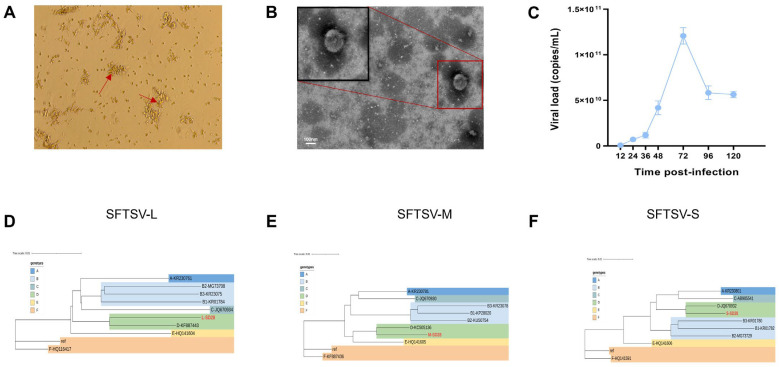
Isolation, propagation and genetic characterization of the SFTSV strain used in this study. (**A**) Representative cytopathic effects induced by SFTSV infection in Vero cells, showing clustered, degenerative cell foci (red arrows). (**B**) Transmission electron micrograph of purified SFTSV particles displaying typical enveloped, spherical virions; the boxed region shows a magnified view. Scale bar, 100 nm. (**C**) Growth kinetics of SFTSV in cell-culture supernatants as determined by quantitative RT-PCR, presented as viral RNA copies per mL at the indicated time points post infection. Data are shown as mean ± SD of replicate measurements. (**D**–**F**) Phylogenetic analysis of the L (**D**), M (**E**) and S (**F**) genome segments based on nucleotide sequences. The SFTSV strain used in this study (red box) is highlighted, confirming its close relationship with previously reported SFTSV isolates in all three segments.

SFTSV RNA was detected in serum samples collected during the acute phase of patients using qRT-PCR, with a Ct value of 28.6, indicating a high viral load. The serum was inoculated into Vero cells and subjected to three rounds of blind passage, after which typical virus-induced cytopathic effects (CPE) were observed in the third round of cells, including cell contraction and increased intercellular gaps. Typical viral particles were observed under transmission electron microscopy (Figure 1A,B). The culture supernatant tested positive for viral RNA via qRT-PCR, with the maximum viral load reaching 1.21 × 10^11^ copies/mL, indicating successful replication and stable amplification of the virus in vitro (Figure 1C).

Total RNA was extracted from this viral isolate and subjected to whole-genome sequencing, resulting in high-coverage, high-quality sequences of the three segments of the viral genome: the L segment at 6368 nt, the M segment at 3378 nt, and the S segment at 1744 nt. Alignment of the assembled sequences with the reference strain (L segment GenBank accession: HQ141595; M segment GenBank accession: HQ141596; S segment GenBank accession: HQ141597) showed nucleotide homologies of 95.9%, 95.6%, and 95.4%, respectively, suggesting that the isolate is highly related to the circulating strain, with about 4.1–4.6% sequence divergence (Figure 1D–F).

### 3.2. The Preprocessing of Total RNA Successfully Yields High-Quality Negative-Strand Viral RNA Nanopore DRS Results

**Figure 2:**
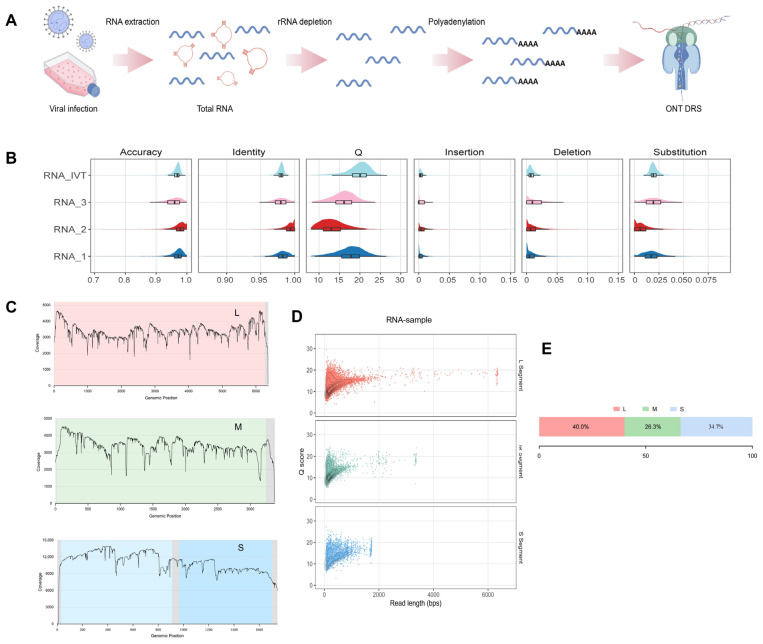
Nanopore direct RNA sequencing of SFTSV-infected cells and quality metrics of virus-mapping reads. (**A**) Schematic overview of the experimental workflow. Cells infected with SFTSV were harvested for total RNA extraction, followed by rRNA depletion and enzymatic polyadenylation of viral RNAs before Oxford Nanopore Technologies (ONT) direct RNA sequencing (DRS). (**B**) Read metrics of sample and IVT reads basecalled using the Dorado basecaller. Read-level accuracy, identity, insertion, deletion, and substitution were calculated based on the mapping results, while the Q score was plotted using all reads. (**C**) Genome-wide coverage profiles of SFTSV L, M and S segments derived from DRS data, showing continuous coverage along each segment with localized drops and enriched regions. (**D**) Relationship between read length and Q score for SFTSV L, M and S reads, shown as scatter and contour density plots, suggesting stable basecalling performance over a broad length range. (**E**) Proportions of SFTSV-mapping reads assigned to the L, M and S genomic segments, suggesting balanced representation across the tripartite genome.

Since SFTSV is a negative-strand RNA virus, its genome naturally lacks the typical poly(A) tail of eukaryotic mRNA, making direct use of DRS technology not feasible. To overcome this limitation, a tail is enzymatically added to the 3′ end of the viral RNA prior to sequencing to facilitate efficient adapter ligation and nanopore loading. Additionally, given the low proportion of viral mRNA in total RNA, the library preparation process typically involves the removal of high-abundance non-coding RNA, such as host and viral rRNA, to enrich for target mRNA transcripts. These strategies are crucial for enhancing the capture efficiency and data validity of viral RNA, serving as a key prerequisite for direct transcriptomic analysis of SFTSV (Figure 2A).

We obtained up to 5,490,688 reads from infected cells, aligning long reads using minimap2 and restricting the analysis to viral genome segments (L, M, and S), with 4.14% of the reads belonging to SFTSV (Table 1). Among these, reads with a Q score ≥20 that aligned to the viral genome exhibited distribution percentages of 40.0% for the L segment, 26.3% for the M segment, and 34.7% for the S segment (Figure 2E). We further successfully analyzed the coverage characteristics of the three genome segments of SFTSV. Our sequencing results indicate full-length coverage for all segments, suggesting that RNA processing and library preparation strategies, including host rRNA removal and tailing, delivered stable and high-quality viral RNA signals. There are distinct coverage patterns among segments: the L segment showed uniform coverage with slight fluctuations at both ends, indicating sufficient sequencing depth for long fragments with statistical power to detect 5′/3′ terminal regions and potential local stalling points. The M segment exhibited stable coverage troughs at multiple locations, suggesting possible low-flux windows or secondary structure-related sequencing accessibility issues in this segment. The S segment showed overall high coverage with sharp declines at specific locations, which correlates with its bidirectional coding (NP and NSs) and is consistent with its transcription/replication balance and structural layout (Figure 2C).

Based on these findings, we further analyzed the relationship between the Q scores and read lengths, discovering that for all three transcripts, nearly full-length reads can be obtained close to their reference lengths. We obtained 30 full-length transcripts of the L segment, 38 of the M segment, and 79 of the S segment. The L segment exhibited the longest read lengths overall, the S segment the shortest, and the M segment was intermediate, consistent with the order of the genome segment lengths, illustrating that DRS maintained a reasonable length hierarchy for viral transcripts (Figure 2D). These results suggest that the artificial poly(A) tailing strategy was adequate for downstream DRS library preparation, as near full-length reads were recovered across all three SFTSV genome segments. However, the average Q score for the L segment was slightly lower than those for the M and S segments, suggesting higher signal noise for longer fragments in the nanopores (Figure 2D). This is consistent with established benchmark studies and recent systematic evaluations of the ONT platform’s accuracy. The quality of sequencing data in this study meets the requirements for downstream analysis. Moreover, the accuracy density of all reads shows a unimodal distribution at the high accuracy end (peak around 0.95–0.98), suggesting high consistency for most sequencing reads and supporting the feasibility of downstream transcript and modification analysis (Figure 2B).

### 3.3. Nanopore DRS Identifies Complex Transcriptome Features of the Negative-Strand RNA Virus SFTSV

**Figure 3:**
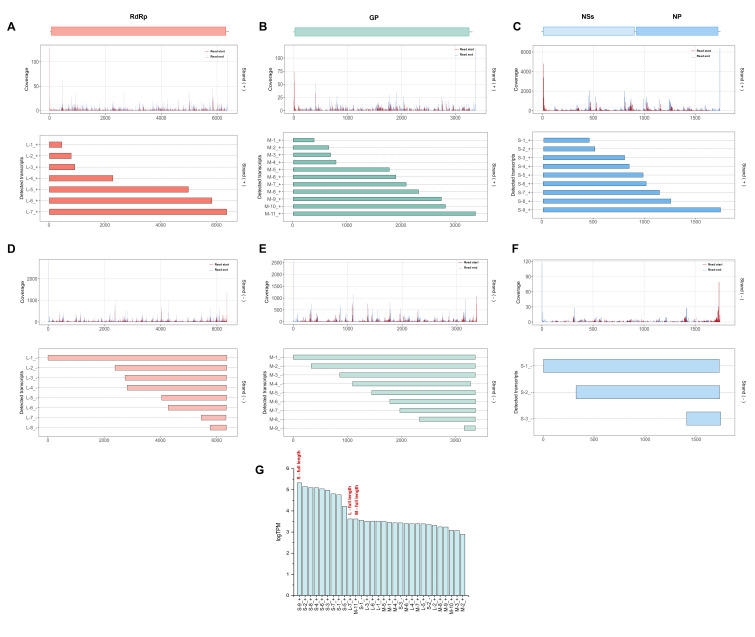
Landscape of SFTSV segment-specific transcription revealed by Nanopore direct RNA sequencing. (**A**–**F**) Genome-wide distribution of read start (blue) and read end (red) positions for the L (RdRp) (**A**,**D**), M (GP) (**B**,**E**) and S (NP/NSs) (**C**,**F**) segments. For each segment, coverage and termini peaks are shown separately for viral (+)RNA (**A**–**C**) and (−)RNA (**D**–**F**), illustrating discrete clusters of transcription initiation and termination along the coding regions. (**G**) Bar plot of transcript abundance (logTPM) for SFTSV-derived transcripts, sorted from highest to lowest. Full-length transcripts from the S, M, and L segments are indicated in red.

DRS enables the acquisition of full-length viral RNA, including genomic and subgenomic transcripts, without the need for reverse transcription. This capability helps to reduce library biases while resolving the transcriptome composition and dynamics. Based on strand-specific alignment and end-point clustering, we obtained transcription maps of the three SFTSV segments (L, M, and S), showing that the coverage profiles and read start-end positions in the samples align with each other, supports the overall consistency of the dataset (Figure 3A–C). The L segment predominantly consists of full-length RdRp transcripts, alongside several medium and short-length categories whose start and end coordinates align spatially with the end-point peaks. This pattern is consistent with reproducible transcript boundaries and is less suggestive of diffuse degradation alone. The M segment is the richest in truncated forms, with detected transcripts spread widely and interspersed along the horizontal axis, ranging in length from nearly full-length to various medium and short categories, partially overlapping yet converging at distinct endpoints. In the S segment, NP and NSs signals are clearly separated by polarity, forming multiple length categories within each polarity, with transcript bars distinctly divided into two groups: one covering NP and another covering NSs. These groups partially overlap on the genome but have separate endpoint coordinates, consistent with the ambisense coding organization of the S segment (Figure 3D–F). Expression abundance analysis of the segments shows that a few full-length transcripts dominate the primary expression share of each segment, while length categories defined by pairs of end-point clusters constitute a stable secondary tier. After log transformation, rank-ordered transcript abundance (logTPM) displayed a characteristic long-tailed distribution: a small subset of highly abundant transcripts accounted for most expression, whereas numerous variants clustered in the low-abundance range, suggesting pronounced abundance stratification. Notably, full-length transcripts from all three genome segments were enriched among the higher-abundance tier, suggesting that canonical-length products predominate in infected cells while multiple low-abundance transcript variants coexist. Overall, the data support a tiered length spectrum model anchored by discrete termination hotspots, suggesting that these RNA species are unlikely to be explained solely by random truncation and may instead represent reproducible transcript categories associated with viral transcription Overall, these data support a tiered length-spectrum model anchored by discrete endpoint hotspots. The recurrent distribution of transcript lengths around these hotspots suggests the presence of reproducible RNA species, although independent experimental validation will be required to determine whether they arise from regulated transcriptional termination rather than technical or degradation-related processes.

To further evaluate the robustness of the observed transcriptomic patterns, we assessed concordance across the three biological replicates using transcript-level TPM values. Pairwise correlation analysis showed moderate-to-high agreement between replicates (Spearman ρ = 0.740–0.763; Pearson r = 0.849–0.976) (Figure S2A,B). At the segment level, the abundance hierarchy was preserved across all replicates, with the S segment consistently showing the highest total TPM, followed by M and then L (Figure S2C). Although some inter-replicate variation was observed, the overall abundance structure remained stable, suggesting that the major transcriptomic patterns reported here are unlikely to be explained solely by sequencing-related technical variation.

### 3.4. Nanopore DRS Reveals Potential Modification Sites in the Transcriptome of SFTSV, a Negative-Strand RNA Virus

To comprehensively and accurately identify natural modification sites on SFTSV RNA, we utilized nanopore current signal analysis tools, implementing IVT RNA as an unmodified control (Figure S3). This established a highly reliable system for viral epitranscriptome identification. The IVT samples serve as an ideal “demodification background” due to the lack of participation by RNA modification enzymes, effectively filtering out false-positive signals caused by secondary structures, sequencing noise, or algorithm biases during sequencing. By comparing the modification probabilities at each site between SFTSV and its IVT samples, we identified the actual presence and segment-specific distribution of three types of modifications (m6A, m5C, Ψ) on SFTSV transcripts.

**Figure 4:**
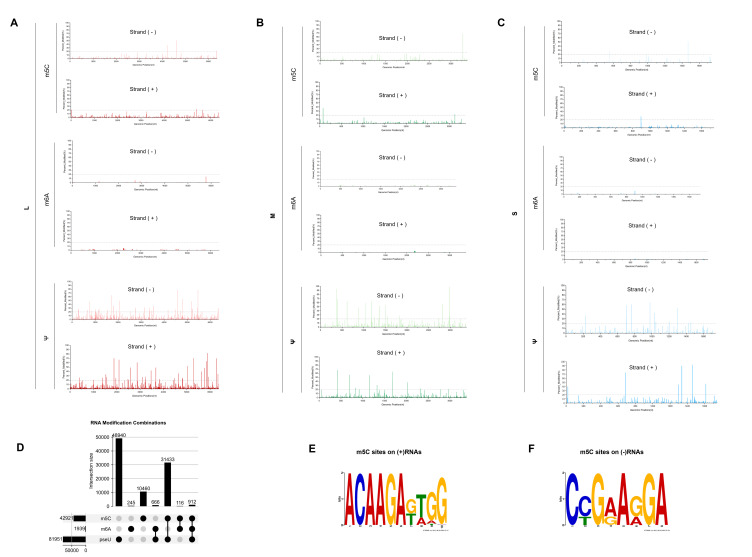
Segment- and strand-resolved landscape of SFTSV RNA modifications and associated sequence context. (**A**–**C**) Genomic distribution of three RNA modification types—5-methylcytidine (m^5^C), N6-methyladenosine (m^6^A) and pseudouridine (Ψ)—across the L (**A**), M (**B**) and S (**C**) segments. For each segment, modification signals are shown separately for negative-sense (viral genomic) and positive-sense (viral mRNA/antigenomic) strands as a function of genomic position, illustrating discrete hotspots and strand-biased enrichment patterns. (**D**) Combinatorial usage of m^5^C, m^6^A and Ψ on SFTSV RNAs. The bar plot indicates the number of sites assigned to each modification combination, and the dot matrix below denotes the corresponding modification sets, highlighting the predominance of singly modified sites and the presence of loci harboring multiple modification types. (**E**,**F**) Sequence logos depicting the nucleotide context of high-confidence m5C sites on viral (+)RNAs (E) and (−)RNAs (**F**), revealing distinct motif preferences associated with m5C installation on different RNA species.

We scanned the entire genome of the three SFTSV segments (L, M, S) for potential RNA modification sites and summarized their spatial distribution on the negative strand (Strand−) and positive strand (Strand+). Overall, the three types of modifications exhibit a significant abundance gradient, with Ψ being the most abundant, followed by m5C, and m6A being the least abundant and sparsely distributed. This pattern is consistently reproducible across the L, M, and S segments. In strand-specific distribution, m^5^C and Ψ show higher peak density and amplitude on the negative strand, while the positive strand predominantly features scattered peak clusters. m6A is observed only in a few isolated peaks on both strands, without forming obvious enrichment zones, and the modification probabilities remain below the 20% threshold. Comparative analysis among segments shows that the L segment has the widest modification range, with Ψ forming multiple clusters on both strands and m^5^C enriched in a band-like scatter on the positive strand; the M segment shows overall lower peak density than L but retains the relative relationship of “Ψ > m^5^C ≫ m^6^A”; the S segment, although shortest, presents distinct high-peak areas of Ψ and m5C on the positive strand, with only a few scattered peaks on the negative strand (Figure 4A–C). Additionally, we systematically summarized locus-level results for the three genome segments, listing the coordinates of each modification on different segments and strands, detailed in Supplementary Table S3.

Analyzing the combinatorial relationship of the three RNA modifications (m^5^C, m^6^A, Ψ) using individual reads as statistical units reveals that most modified reads carry only one type of modification, with “Ψ only” being the most prevalent and “m^5^C only” following. Multi-modification events (with the same read carrying ≥2 types of modifications) are relatively rare, with the Ψ + m^5^C combination being the most common; reads containing m6A are relatively few, and reads co-occurring with other modifications are also rare; the triple combination (m^5^C + m^6^A + Ψ) accounts for only a minimal proportion. These findings suggest that at the single-molecule level, modifications primarily manifest as “single-modification reads,” with multi-modification events being in the minority; Ψ and m5C form the main background, whereas m6A labeling is confined to a limited number of specific reads (Figure 4D). Sequence motif analysis further shows that m5C modifications on viral (+) RNA tend to occur in upstream sequences enriched in A/C, while m5C modifications on (−) RNA are more skewed towards C/G rich environments with adjacent multi-C sequences, reflecting significant context-dependent differences (Figure 4E,F).

### 3.5. Modification Features of Specific SFTSV Transcripts

**Figure 5:**
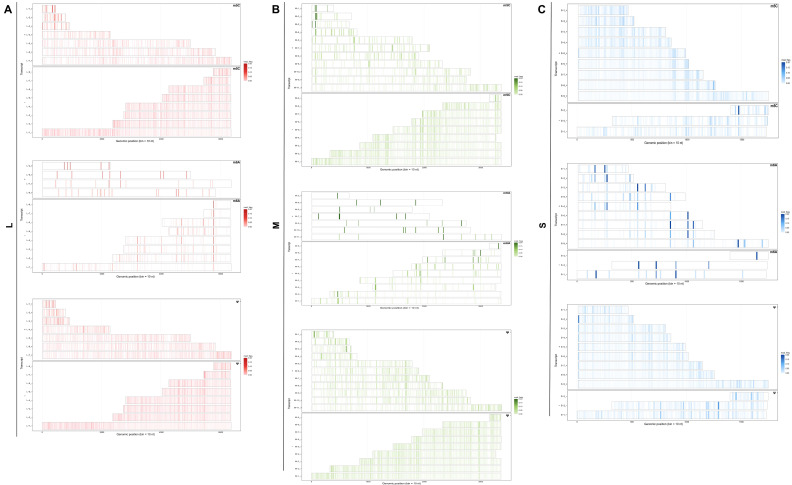
Transcript-resolved maps of RNA modifications across the SFTSV genome (**A**–**C**). For each genomic segment—L (**A**), M (**B**) and S (**C**)—rows represent individual viral transcripts (including (+)RNA and (−)RNA isoforms), and columns are genomic positions binned at 10 nt resolution. Vertical colored tiles mark bins harboring modification calls; color intensity encodes the per-bin modification frequency (mod_freq). Gray boxes highlight representative hotspots. Within each segment, panels (top to bottom) show m^5^C, m^6^A, and Ψ (pseudouridine). Across segments, Ψ is the most prevalent mark, m^5^C is intermediate, and m6A is sparse. Hotspots align across transcripts at the same genomic coordinates, suggesting locus-driven modification.

To systematically compare RNA modification patterns across the three SFTSV genomic segments under different transcript contexts, we mapped the identified m5C, m6A, and Ψ sites back to their corresponding transcript isoforms and constructed transcript-resolved epitranscriptomic topological maps for the L, M, and S segments (Figure 5A–C). Despite pronounced transcript-length heterogeneity across all three segments, modification positions did not show obvious positional shifts among isoforms as transcript length varied. Instead, whenever long and short (subgenomic) transcripts spanned the same genomic region, modification hotspots were reproducibly observed at the corresponding positions, appearing as clear vertically aligned patterns across isoforms in the visualization. This feature was consistently observed for m5C, m6A, and Ψ, suggesting cross-isoform positional conservation of SFTSV RNA modification sites. These observations further suggest that modification deposition is unlikely to be random and is more likely constrained by local sequence context and/or cis-acting features, consistent with strong site selectivity.

**Figure 6:**
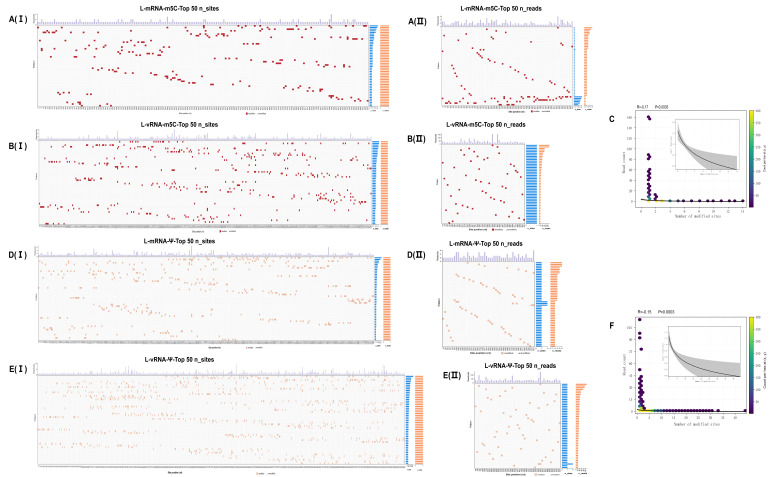
Landscape of base-modification pattern combinations in the L transcript. Panels (**A**,**B**) (m^5^C) and (**D**,**E**) (Ψ): Heatmaps summarize modification pattern combinations for the L transcript in viral (+) RNA and (−) RNA. For each RNA type, two views are shown: (I) Top 50 by number of modified sites per read pattern (n_sites) and (II) Top 50 by read support (n_reads). Rows correspond to unique read patterns; rows are ordered from top to bottom by the ranking metric (n_sites in I; n_reads in II). Columns represent genomic positions (only positions that show at least one modified call among the Top 50 rows are retained). Colored squares indicate modified calls in a read at a given position (red for m^5^C; light orange for Ψ); light gray indicates unmodified; white indicates missing. The top bar above each heatmap gives the per-site modified-read load (total reads supporting modification at that position, computed from the rows shown). On the right, two horizontal bar strips provide per-pattern summaries: blue = number of modified sites (n_sites) and orange = read support (n_reads). Panels C and F: Relationship between the number of modified sites per pattern and the read support for m^5^C (**C**) and Ψ (**F**). Each point is a read pattern; the color scale reflects site density (or count of patterns). The inset shows a non-parametric smooth (LOESS) with 95% confidence ribbon. Reported statistics denote the correlation between n_sites and n_reads (C: R = −0.17, *p* = 0.0003; F: R = −0.16, *p* = 0.0003).

We systematically characterized the combination patterns of m5C and Ψ on SFTSV (+) RNA and (−) genome RNA across the three segments (Figure 6, Figure S4 and Figure S5A,B,D,E). To avoid dilution of information from low-abundance patterns and absent sites, each heatmap displays only the Top 50 rows, sorted by the number of modification sites contained (n_sites) and the read support (n_reads) for each reading pattern. Consistent organizational patterns are observed across the three segments: regardless of whether it is (+) RNA or (−) RNA, the modification distribution shows a significant long-tail characteristic, with the vast majority of “patterns” containing only 1–2 modification sites, while only a few patterns include more sites. In terms of read support, there is a coexistence of “few high, many low.”

Comparing (+)RNA to (−) RNA, all three segments show that (−) RNA modifications are more dispersed across genomic coordinates but have sharper peak loads, suggesting more concentrated “hotspot” sites on the genomic template; conversely, (+) RNA is characterized by multiple moderate-load sites. Further quantifying the relationship between the number of modification sites per pattern (n_sites) and their read support (n_reads) reveals a weak negative correlation for both m5C and Ψ (m^5^C: R = −0.17, *p* = 0.0003; Ψ: R = −0.16, *p* = 0.0003), with the distribution strongly skewed to the right. Most patterns have few sites and low support, with a few patterns contributing the majority of reads. LOESS smoothing (inset) similarly shows a rapid decline in low site number regions, followed by a plateau (Figure 6, Figure S4 and Figure S5C,F). Overall, for these three segments, “being abundantly detected” is more dependent on the presence of a few high-load sites rather than the accumulation of a large number of sites within a single pattern

## 4. Discussion

Once a virus infects a host, the process of its gene expression becomes complex, involving a multitude of regulatory mechanisms [37,38]. In this study, we applied DRS to native SFTSV RNA from infected cells and jointly analyzed transcript architecture and RNA modification signals. Our findings should not be interpreted as the first evidence of RNA modification on SFTSV RNA, because previous studies have already implicated m6A and host m6A machinery in SFTSV infection. Instead, the main advance of this work is the integrated, strand-aware, and isoform-resolved characterization of the SFTSV transcriptome and epitranscriptome in infected cells. DRS thus provides a useful framework for studying transcript diversity and candidate modification patterns in intracellular viral RNA.

Unlike eukaryotic mRNAs, the genome of SFTSV, a negative-sense RNA virus, lacks a native poly(A) tail [39,40]. DRS platforms depend on poly(A) tracts for RNA capture and library construction; hence, enzymatic polyadenylation is required before library preparation. This process appends a synthetic poly(A) tail, allowing adapter ligation and workflow compatibility [33,41,42]. Additionally, host-derived ribosomal RNAs are abundant in samples. When viral replication is active yet viral mRNA copies are comparatively low, rRNA can vastly outnumber viral transcripts [43]. Therefore, depleting host rRNA before library preparation is essential to enhance sequencing coverage of viral transcripts. This optimized workflow improved the recovery of native SFTSV RNA and supported downstream analyses of both transcript organization and candidate RNA modifications. The resulting long-read dataset enabled segment-resolved and strand-specific characterization of SFTSV transcriptional structure, while also allowing modification-associated signals to be examined on the same native RNA molecules. We observed that the reads predominantly fall within a primary density range of Q≈10–20 with an accuracy of 0.95–0.98, aligning with empirical performance expectations of nanopore platforms, ensuring the data quality is suitable for high-resolution endpoint modeling. Notably, the sequencing data support strong strand specificity, offering a robust foundation for determining transcriptional orientation and termination sites of the negative-sense virus.

At the transcript level, our DRS data revealed substantial structural complexity in SFTSV RNAs during host-cell infection. A major feature of the dataset was a length-stratified transcriptional hierarchy organized around discrete 3′ termination hotspots. A limited number of full-length transcripts accounted for most of the expression signal, whereas multiple shorter transcripts formed a lower-abundance tier. These findings suggest that SFTSV transcription is not simply represented by one dominant RNA species per segment, but instead consists of a reproducible set of endpoint-defined transcript classes. Such a transcriptional organization may reflect suggestive of regulated termination or polymerase pausing events less consistent with random degradation alone [44,45]. Notably, although SFTSV is a segmented negative-sense RNA virus lacking alternative splicing, we still detected extensive variation in transcript lengths. These non-full-length transcripts may originate from template secondary structures, occupancy by RNA–protein complexes, and polymerase pausing or reinitiation at specific loci. This is consistent with the stable peak–valley coverage pattern observed across SFTSV’s three segments. Previous studies on the Orthobunyavirus Bunyamwera virus have demonstrated frequent 3′-end premature termination and truncated mRNAs when translation is uncoupled, whereas coupling to host translation attenuates this effect, underscoring the necessity of transcription–translation coupling for generating full-length mRNAs [46]. Furthermore, in the nonsegmented negative-sense RNA virus RSV, Nanopore DRS has uncovered polycistronic read-through mRNAs and heterogeneous poly(A) tail lengths, revealing the complexity of its RdRp in intergenic transcription and termination control. However, this interpretation should be made with caution. Although the recurrence of discrete endpoint clusters across replicates and genome segments is consistent with non-random transcript organization, the present evidence is derived primarily from nanopore sequencing and bioinformatic reconstruction. As such, we cannot fully exclude potential contributions from library-preparation artifacts, partial RNA degradation, or length-dependent capture biases inherent to long-read direct RNA sequencing. Therefore, while our data support the existence of reproducible truncated transcript species and are compatible with regulated transcriptional termination, they do not by themselves constitute direct experimental proof of such a mechanism. Independent validation using approaches such as transcript-end RT-PCR, Northern blotting, or other transcript-boundary mapping strategies will be important in future studies to confirm the physical existence and precise boundaries of representative isoforms.

Comparative analysis of the three genome segments suggests that their transcript architectures are not identical. The L segment chiefly consists of full-length RdRp transcripts with multiple mid- and short-length species, a pattern that may be compatible with differential control of polymerase-related RNA output. The M segment displays abundant truncation categories and repeatable termination hotspots, suggesting that glycoprotein-related transcripts may have relatively greater termination heterogeneity than those of the other segments. Polarity-resolved analysis of the S segment shows near-full-length species in the NP direction but denser hotspots in the NSs direction. This asymmetry may be relevant to the ambisense organization of the S segment, although its functional significance remains to be determined. Overall, these observations suggest that transcript-end distribution may represent an important layer of SFTSV transcriptional organization. We also observe non-uniform expression among segments; the S segment is generally more highly expressed. This pattern is broadly consistent with the functional importance of the proteins encoded by the S segment. Previous studies have suggested that NSs contributes to interferon antagonism and that segmental expression may vary across the course of infection. In this context, the segment-dependent abundance patterns observed here may provide a useful descriptive framework for future studies examining how transcript organization relates to viral replication and host interaction.

Relative to earlier transcriptome studies based on short-read sequencing, Illumina-based profiling may not fully resolve the structural diversity of viral RNAs; particularly with respect to 5′- and 3′-truncated isoforms, which may limit detailed analysis of transcript boundary variation and its relationship to promoter usage, transcriptional termination, or RNA stability. In contrast, the full-length native RNA reads provided by DRS allow direct detection of these structural transcript variants, thereby providing a framework for more detailed characterization of viral RNA architecture in infected cells.

At the epitranscriptome level, our nanopore DRS data enabled integrated mapping of candidate m^6^A, m^5^C, and pseudouridine signals on native SFTSV RNAs across the L, M, and S segments and on both positive- and negative-sense strands. Overall, the observed segmental hierarchy, strand asymmetry, and predominance of single-modification reads suggest that the SFTSV epitranscriptome is organized in a non-uniform manner. However, the functional consequences of these patterns should be interpreted cautiously. Rather than demonstrating direct regulatory roles, the present data provide a transcript-resolved framework that raises the possibility that different modification types may contribute in distinct ways to viral RNA stability, immune recognition, or expression efficiency during infection.

Throughout the SFTSV genome, all three modifications are detectable, each with a distinct spatial distribution. Ψ is broadly and continuously distributed across the segments and strands; correspondingly, most modified reads contain a single modification, usually Ψ. Prior research shows that replacing uridine with Ψ significantly reduces TLR3/7/8 and PKR activation while enhancing mRNA translation and stability, thereby lowering immune detection and increasing output [47,48]. Therefore, the Ψ-dominant pattern observed here is compatible with the possibility that this modification may contribute to shaping the intracellular behavior of SFTSV RNA. However, such functional implications remain hypothetical in the absence of direct perturbation experiments. For m5C, discrete peaks appear on all segments, often enriched on the negative strand; m5C motifs differ between (+) RNA and (−) RNA, suggesting context-dependent variation. Given previous findings that NSUN2-catalyzed m5C dampens type I IFN responses and enhances RNA stability/replication across various viruses, it is plausible that (−) RNA during replication and (+) RNA during expression recruit distinct NSUN paralogs/cofactors or assume different structural states, leading to strand- and segment-specific enrichment and motif divergence [49,50]. At present, however, this remains an inference based on distributional patterns and published knowledge of m5C biology rather than a direct functional conclusion from the current dataset. Although m6A occurs at low abundance, falling below detection thresholds, SFTSV may possess few but functionally critical m6A sites, shown by previous studies that report potential m6A sites in SFTSV, demonstrate that the m6A reader YTHDF1 restricts SFTSV replication, and suggest that SFTSV recruits m6A regulators to enhance infection. This underscores the necessity for orthogonal validation of RNA modification calls [51,52].

Additionally, at single-transcript resolution, the three SFTSV segments (L/M/S) showed a broadly similar modification pattern, dominated by Ψ and m5C, with comparatively sparse and scattered m6A signals. Most hotspots were aligned at similar genomic coordinates across co-linear transcripts, suggesting that modification-associated signals may be more strongly constrained by genomic position than by transcript-specific abundance alone. We also observed unmodified reads in some transcripts, heterogeneity in modification occupancy against this position-biased background, which might also reflect coverage and temporal resolution limitations [53]. The presence of aligned hotspot bands across transcripts within each segment raises the possibility that these modification patterns may reflect an interplay between replication/transcription-associated processes and local sequence or structural context, rather than being explained solely by transcript abundance. In bunyaviruses, the L protein is responsible for genome replication and transcription, initiating transcription by acquiring host-capped fragments via the cap-snatching mechanism. This process has been elucidated and validated experimentally in the overall conformation of SFTSV and its N-terminal endonuclease structure [54,55]. It is therefore possible that differences in polymerase behavior during initiation, pausing, or template switching, together with local termination-related signals, may contribute to the recurrent hotspot-like patterns observed at specific positions [56,57]. In this context, interaction between recurrent hotspot regions and the sequence or structural preferences of host-modifying enzymes, such as PUS family proteins or NSUN-related methyltransferases, may offer one possible explanation for the co-located modification hotspots and segment-specific spatial heterogeneity observed here [24,25,50,58]. However, this interpretation remains inferential and will require direct experimental validation.

At single-molecule resolution, we further characterized the combinatorial organization of two major candidate RNA modification types, m5C and Ψ, across SFTSV transcripts. These modifications are organized in a “sparse-yet-focal” manner on both viral (+) RNA and (−) RNA. Most transcripts contain only one or two modified sites, but some have high-load hotspots at specific genomic locations, reflecting a long-tailed distribution. This pattern is broadly consistent with previous observations from ONT DRS studies and with comparative statistical frameworks such as Nanocompore used to evaluate RNA modification-related signal variation [59,60]. When examining the correlation between the number of modified sites (n_sites) and read counts (n_reads), we found only a weak negative relationship. This suggests that high detection counts are largely attributed to a few heavily loaded modification sites, rather than the overall number of modified sites. Such observations are in harmony with the known roles of RNA modifications, Ψ enhances mRNA stability, translation, and diminishing immune detection via PKR/TLR pathways, while m5C influences RNA stability, nuclear export, and translational efficiency [47,61,62,63]. In this context, the hotspot-dominant distribution observed here is compatible with the possibility that site-specific modification burden, rather than modification number alone, may be biologically relevant. Accordingly, the local context and occupancy of modification hotspots may be more informative than modification number alone. Interestingly, (+) RNA and (−) RNA exhibit distinct modification patterns. (−) RNA modifications are more dispersed but have sharper intensities at specific coordinates, whereas (+) RNA modifications tend to spread over multiple moderately loaded sites. These differences may reflect distinct constraints acting on viral RNA synthesis, potentially involving local RNA secondary structure, cofactor environment, or polymerase-associated processes [64]. One testable hypothesis raised by this hotspot-dominant distribution is that a limited number of highly occupied sites may disproportionately influence local RNA architecture or polymerase–template interactions, potentially contributing to differences in read abundance or transcript boundary usage [58,65]. However, this interpretation remains inferential and will require future functional validation.

Building on previous studies of RNA modifications in SFTSV, the present work provides an integrated view of transcript architecture and multiple candidate RNA modification patterns in infected cells through direct sequencing of native RNA molecules. Our findings expand the descriptive framework for understanding RNA length heterogeneity, transcript-end distribution, and modification landscape organization in SFTSV RNA, while also highlighting the utility of Nanopore DRS for resolving transcriptional detail and candidate RNA modification signals at single-molecule resolution. Importantly, this study primarily provides a strand-aware, isoform-resolved analytical framework rather than direct functional evidence for the effects of individual modification sites. The precise roles of these candidate sites, and their relevance to viral infection, will require further orthogonal validation and mechanistic investigation. Overall, this work offers a useful data resource and technical reference for future studies of SFTSV RNA biology and the possible relationship between RNA modification and virus–host interaction.

## Figures and Tables

**Table 1:** Raw SFTSV read features and mapping statistics based on Dorado.

	RNA_1	RNA_2	RNA_3
Average percent identity	96.4	93.9	96.1
Fraction of bases aligned	0.9	0.9	0.9
Mean read length	382.0	528.5	510.3
Mean read quality	15.2	12.1	14.3
Median percent identity	97.2	94.0	96.7
Median read length	306.0	380.0	368.0
Median read quality	17.9	13.2	16.2
Number of reads	149,584.0	12,574.0	227,261.0
Read length N50	467.0	654.0	318.0
Total bases	57,140,535.0	6,644,907.0	59,881,881.0
Total bases aligned	52,681,901.0	5,999,973.0	53,227,517.0

## Data Availability

The data presented in this study are deposited in the China National Center for Biotechnology (CNCB) as BioProject ID: PRJCA051537, GSA Accession: CRA033781.

## Supplementary Materials

The following are available online at https://www.mdpi.com/article/10.3390/microorganisms14040756/s1, Figure S1: Schematic representation of the SFTSV tripartite genome and primer amplicons used for in vitro transcription,; Figure S2: Reproducibility analysis of transcript abundance across biological replicates; Figure S3: Coverage profiles of the three SFTSV genomic segments in the IVT sample; Figure S4: Pattern combinations of m^5^C and Ψ modifications on the M transcript; Figure S5. Pattern combinations of m^5^C and Ψ modifications on the S transcript, Table S1: SFTSV in vitro transcription primer; Table S2: Consensus termination-site families (TES) and associated start-site subtypes (TSS); Table S3: Sequencing data of modification sites.

## Abbreviations

The following abbreviations are used in this manuscript:

SFTS	Severe Fever with Thrombocytopenia Syndrome
SFTSV	Severe Fever with Thrombocytopenia Syndrome Virus
DRS	direct RNA sequencing
NSs	non-structural protein
NP	nucleoprotein
GP	glycoprotein precursor
RdRp	RNA-dependent RNA polymerase
m^6^A	N6-methyladenosine
m5C	5-Methylcytosine
pseU	Pseudouridine
