# Supplementary material for: Transcriptomic and RNA Modification Landscape of Severe Fever with Thrombocytopenia Syndrome Virus Revealed by Nanopore Direct RNA Sequencing
Source: Microorganisms. 2026 Mar 27;14(4):756. doi: 10.3390/microorganisms14040756 (PMC13118471; doi:10.3390/microorganisms14040756)
Supplement: Supplementary file 1 [file microorganisms-14-00756-s001.zip › Supplement figures.pdf]

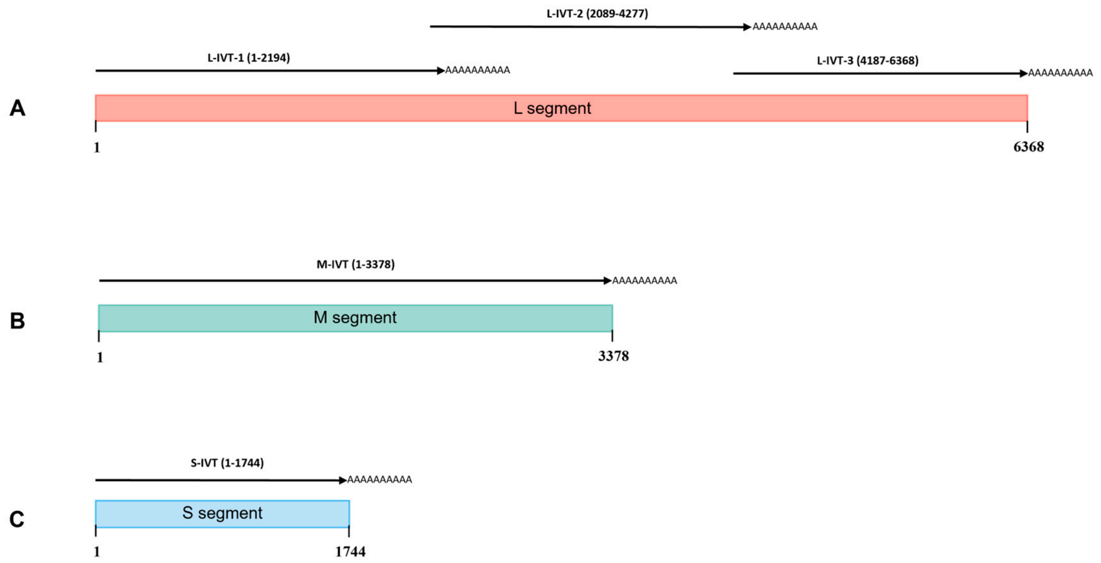

Figure S1. Schematic representation of the SFTSV tripartite genome and primer amplicons used for in vitro transcription.

The negative-sense RNA genome of severe fever with thrombocytopenia syndrome virus (SFTSV) consists of three segments, L (large), M (medium) and S (small), depicted as colored horizontal bars proportional to their nucleotide lengths. Black line segments above each bar indicate the genomic regions amplified by specific primer pairs to generate DNA templates for in vitro transcription, thereby producing segment-derived RNA fragments used in downstream assays.

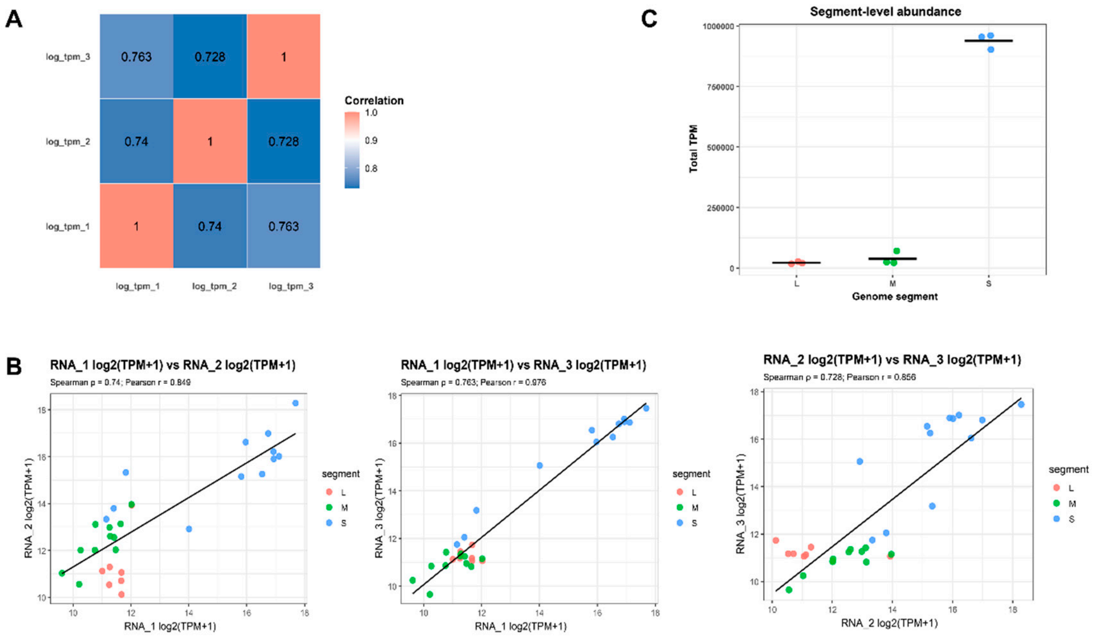

Figure S2. Reproducibility analysis of transcript abundance across biological replicates.

(A) Heatmap of Spearman correlation coefficients among the three biological replicates based on transcript-level log2(TPM + 1) values. (B) Pairwise scatter plots of transcript abundance among the three biological replicates based on log2(TPM + 1) values. Each dot represents one transcript and is colored by genome segment (L, M, or S). Black lines indicate linear regression fits, and Spearman and Pearson correlation coefficients are shown in each panel. (C) Segment-level total TPM values across the three biological replicates. Each dot represents one biological replicate, and black horizontal bars indicate the mean for each segment.

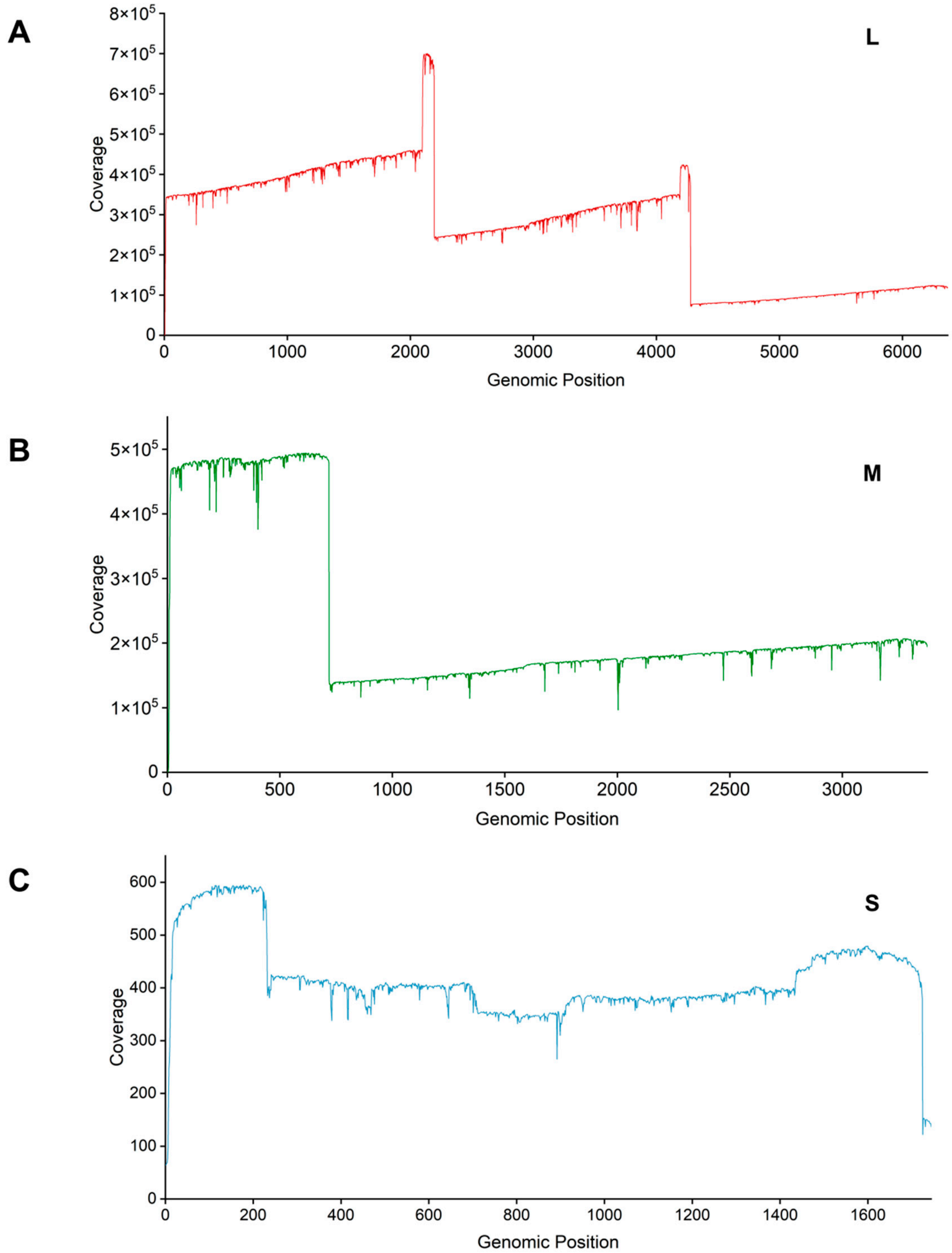

Figure S3. Coverage profiles of the three SFTSV genomic segments in the IVT sample.
(A) L segment; (B) M segment; (C) S segment. The x-axis indicates genomic position, and the y-axis indicates sequencing coverage. The line plots show coverage variation across the full length of each segment in the in vitro transcribed (IVT) sample.

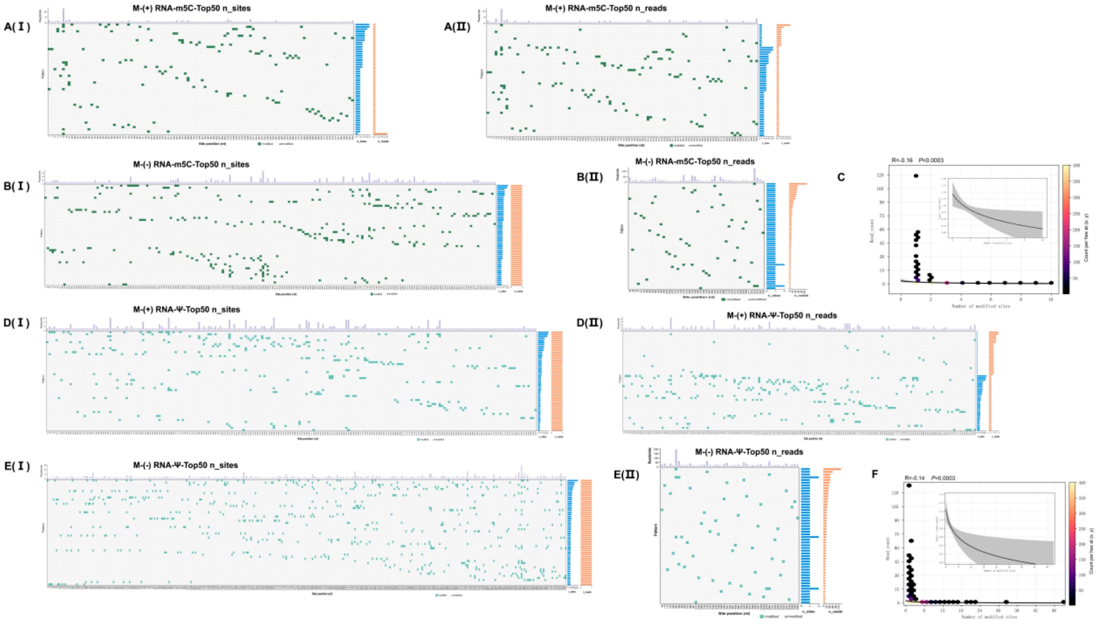

Figure S4. Pattern combinations of m5C and Ψ modifications on the M transcript

Panels A–B (m5C) and D–E (Ψ): Heatmaps depict single-molecule modification pattern combinations for the viral M transcript in subgenomic mRNA (M-mRNA) and genomic RNA (M-vRNA). For each modification and RNA type, two representations are shown: (I) Top 50 read patterns ranked by the number of modified sites per pattern (n_sites) and (II) Top 50 read patterns ranked by read support (read_count). Each row corresponds to a unique read pattern; rows are ordered from top to bottom according to the respective ranking metric (n_sites in I; read_count in II). Columns represent genomic positions along the M transcript; only sites that exhibit at least one modified call within the selected Top 50 patterns are displayed. Filled squares indicate positions called as modified in that pattern (green for m5C, cyan for Ψ); light grey squares indicate unmodified calls; white indicates missing data.

For each heatmap, the top bar plot shows the per-site modified-read load, calculated as the total number of reads supporting modification at that position among the displayed patterns. On the right, two horizontal bar strips summarize for each pattern the number of modified sites (blue; n_sites) and the read support (orange; read_count).

Panel C (m5C) and Panel F (Ψ): Scatter plots show the relationship between the number of modified sites per pattern (x-axis) and the corresponding read support (y-axis) for all M-transcript patterns, with point color indicating pattern density or site count as indicated by the color scale. Insets display LOESS-smoothed fits with 95% confidence intervals. The reported correlation coefficients (C: R = 0.16, *P* = 0.0003 for m5C; F: R = 0.14, *P* = 0.0003 for Ψ) quantify the weak yet significant association between modification multiplicity and read support.

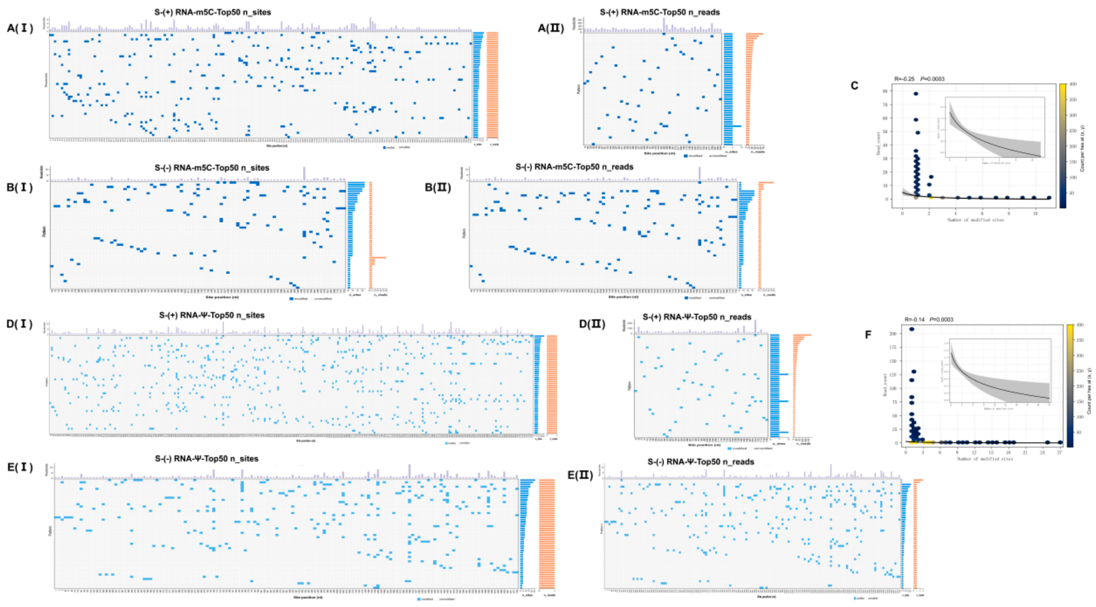

Figure S5. Pattern combinations of m5C and Ψ modifications on the S transcript

Panels A–B (m5C) and D–E (Ψ): Heatmaps display single-molecule modification pattern combinations for the viral S transcript in subgenomic S-mRNA and genomic S-vRNA. For each modification and RNA class, two complementary representations are shown: (I) Top 50 read patterns ranked by the number of modified sites per pattern (n_sites), and (II) Top 50 read patterns ranked by read support (read_count). Each row corresponds to a unique read pattern; rows are ordered from top to bottom according to the respective ranking metric (n_sites in I; read_count in II). Columns represent genomic positions along the S transcript, and only positions that exhibit at least one modified call among the selected Top 50 patterns are shown.

Filled squares indicate positions called as modified in that pattern (dark blue for m5C; light blue for Ψ), light grey squares denote unmodified sites, and white indicates missing data. The top bar plot above each heatmap quantifies the per-site modified-read load, i.e. the total number of reads supporting modification at that position within the displayed patterns. On the right, two horizontal bar strips summarize for each read pattern the number of modified sites (blue; n_sites) and the read support (orange; read_count); these metrics use independent integer-based axes and are separated by a blank spacer to emphasize their distinct scales.

Panel C (m5C) and Panel F (Ψ): Scatter plots illustrate the relationship between the number of modified sites per pattern (x-axis) and read support (y-axis) across all S-transcript patterns. Each point represents a unique pattern; point color reflects pattern density or count as indicated by the color scale. Insets show LOESS-smoothed fits with 95% confidence intervals. The reported correlation coefficients (C: R = 0.25, P = 0.0003 for m5C; F: R = 0.14, P = 0.0003 for Ψ) indicate a statistically significant but overall modest association between modification multiplicity and read abundance on the S transcript.
